# Indirect osteoblast differentiation by liposomal clodronate

**DOI:** 10.1111/jcmm.13366

**Published:** 2017-10-24

**Authors:** Emi Okada, Hidemi Nakata, Maiko Yamamoto, Shohei Kasugai, Shinji Kuroda

**Affiliations:** ^1^ Department of Oral Implantology and Regenerative Dental Medicine Graduate School of Medical and Dental Sciences Tokyo Medical and Dental University Tokyo Japan

**Keywords:** bisphosphonates, soluble RANK, osteoblasts, clodronate liposomes, exosomes

## Abstract

Bisphosphonates impair function of osteoclasts and prevent bone resorption, the mechanism of which has been studied extensively. However, the possible effects of bisphosphonates on chondroblast differentiation and calcium deposition by osteoblasts have only been demonstrated recently. Moreover, cells from monocytic lineage are capable of stimulating osteoblast proliferation. Hence, susceptibility of osteoblasts to various factors requires further investigation. A primary culture of bone marrow‐derived stromal cells was treated with liposomal clodronate (0.1, 0.5, or 1.0 mg/ml) or conditioned medium from liposomal clodronate. Liposomal clodronate (0.25 mg) was injected into mouse femur for *in vivo* experiments. The effects of liposomal clodronate were examined by alkaline phosphatase staining and/or activity assay, and real‐time RT‐PCR was used for studying the effect on osteogenic gene expression. Administration of liposomal clodronate to bone marrow‐derived mesenchymal stromal cell culture enhanced alkaline phosphatase activity and mRNA levels of Runx2 and Dlx5. In addition, conditioned medium from liposomal clodronate also stimulated osteogenic characteristics similar to those of observed *in vitro*, and the number of exosomes in the conditioned medium was highest when pre‐treated with liposomal clodronate. Western blot analysis revealed the presence of RANK proteins in exosomes collected from conditioned medium of liposomal clodronate. Identical observations were obtained *in vivo*, as liposomal clodronate‐injected mouse femur showed increased alkaline phosphatase activity and Runx2 and Dlx5 mRNA expressions, even though the numbers of monocytes and macrophages were reduced. In conclusion, osteoblast differentiation was promoted *via* soluble RANK‐containing exosomes in response to clodronates.

## Introduction

Inflammatory and immune cells, platelets, fibroblasts, osteoprogenitors and mesenchymal stem cells are major participants in the early cascade of bone healing after fracture and in the process of integration of biomaterials with bone tissue. Histological studies of bone revealed that macrophages and multinucleated cells of the monocytic lineage are mobilized to the fractured sites during the early‐phases of inflammation 1. The exact role of these cells during bone healing is not well understood. Several reports on the mechanisms of bone regeneration in acute inflammation models have shown the simultaneous presence of cells of the monocytic lineage and mesenchymal stem cells at the healing sites 2, 3, 4. Monocytes/macrophages are versatile cells that express a wide range of pro‐inflammatory and anti‐inflammatory cytokines, growth and differentiation factors, and chemotactic mediators, which influence bone response, especially osteoblast differentiation, *via* signal transduction involving soluble and insoluble secondary messengers 5. Monocytes/macrophages from mouse spleen can be cocultured with mouse bone marrow cells or MC3T3‐E1/Raw 264.7 cells to stimulate osteoblast differentiation 6, 7. Furthermore, conditioned media from cultured monocytes stimulated osteoblastic proliferation of subsequently cultured MC3T3‐E1 cells 8.

Bisphosphonates are generally applied for prevention against fall or prior to the onset of osteoporosis as they directly inhibit the bone damaging activity of monocytic cells such as osteoclasts. There are several types of bisphosphonates that differ structurally by the presence of a nitrogen molecule. Nitrogenous bisphosphonates such as pamidronate and alendronate inhibit farnesyl diphosphate synthase (FPPS) and thereby block the resorbing activity of osteoclasts at the ruffled border. The non‐nitrogenous bisphosphonates such as clodronates and etidronates are metabolized by osteoclasts, which generates toxic adenosine triphosphate analogues that eventually cause apoptosis 9, 10, 11. The effects of bisphosphonates are still not well defined, although several researchers are concerned about their inflammatory reaction in addition to their inhibitory effects on bone resorption 12. Several recent reports have discussed the effects of bisphosphonates on chondroblast differentiation, suppression of osteoclastic resorption, and calcium deposition by osteoblasts when they are administered locally 13, 14, 15, which is in contrast to their inhibitory effect on monocytic function as activates osteoblasts. Furthermore, the bisphosphonates increase the number of bone marrow mesenchymal stem cells (MBSCs) and promote osteogenesis 16. However, whether local administration of bisphosphonates may induce osteoblast differentiation directly or indirectly and contribute to osteogenesis is not clear. Owing to the complexity of the *in vivo* environment, identification osteoblast differentiation signals induced by locally administered bisphosphonates is a challenging job.

Exosomes are small membrane vesicles of endocytic origin, 30–90 nm in diameter, which correspond to the internal vesicles of multivesicular bodies (MVBs), and are released in the extracellular environment upon fusion of MVBs with the plasma membrane in different cell types 17, 18, 19. They mostly enclose microRNAs, tRNAs and proteins as autocrine or endocrine signal molecules. Exosomes have been detected in a number of human biological fluids such as blood plasma, urine, bronchoalveolar lavage fluid and breast milk, indicating their physiological relevance *in vivo*, and they may even travel distances through biological fluids. Exosomes provide a mode of communication between cells, where one cell can release exosomes that can influence other cells in the same or neighbouring microenvironment, and they trigger immune response, regeneration of dental pulp‐like tissue and wound healing along with activation of angiogenesis 20, 21, 22, 23.

During acute inflammation, monocytes/macrophages are influenced by the surrounding environment within *in vivo* or *in vitro* conditions. Macrophage activation and cytokine and growth factor secretion vary with the nature of the inducing molecule. Although bisphosphonates may contribute to osteoblast differentiation 16, the mechanism may not involve direct stimulation, but an indirect signal transduction to the precursors. Details of the effect of monocyte‐derived mediators on the recruitment and differentiation of mesenchymal cells in response to bisphosphonates are yet to be described. The RANKL‐RANK signalling pathway is a major intercellular stimulation for osteoclastic differentiation 24; however, this signalling cascade is known to operate unidirectionally, from osteoblasts to osteoclast precursors. In this study, RANK expressed in osteoclasts and associated with osteogenesis was investigated as a cargo candidate for exosomes during osteoblast differentiation as we assumed that exosomal signalling pathways may stimulate osteoblastic differentiation. We suggested that monocytes/macrophages and osteoclasts or their precursors secrete signal molecules such as RANK, which is transported in exosomes in response to clodronate, a first‐generation bisphosphonate, resulting in the recruitment and osteogenic differentiation of MSCs.

## Materials and Methods

All animal procedures were approved by the Animal Care and Use Committee of Tokyo Medical and Dental University, Tokyo, Japan (approval #0160198A).

### Cell culture

Adult female Slc: ICR mice (8 weeks‐old) (30 g ± 3 g) were purchased from Sankyo Laboratory services, Inc. (Tokyo, Japan).

Bone marrow‐derived mesenchymal stromal cells (BMSC) were obtained from a mouse femur. The bone shaft was flushed several times with a 25G needle and a 5‐ml syringe containing α‐MEM (α‐MEM 1X: Thermo Fisher Scientific, Waltham, USA), 10% foetal bovine serum (FBS; Merck, Darmstadt, Germany), and a 1% antibiotic‐antimycotic solution (100 × ) containing 10,000 units of penicillin, 10 mg streptomycin and 25 μg amphotericin B per mL (Merck, Darmstadt, Germany). BMSCs were pipetted up and down to disrupt cell aggregates and subjected to centrifugation at 1000 × *g* for 5 min. The cell pellet was resuspended with culture medium, counted and then cultured at an initial density of 3 × 10^5^ cell/well in 12‐well culture plates for further treatment with liposomes.

### Liposomal clodronate or PBS treatment

*In vitro*, liposomal clodronate (LC) or PBS (LP) at different concentrations (0.1, 0.5 and 1.0 mg/ml; ClodronateLiposomes.org, the Netherlands) were added to culture medium supplemented with 10% FBS and 1% antibiotic‐antimycotic solution 12, 13, 14. The control was cultured in α‐MEM without liposomes. After incubation for 12, 24 or 48 hrs, liposomes were carefully washed with PBS, and the medium was switched to an osteoblastic medium containing a cocktail of 1 × 10^−8^M dexamethasone, 50 μg/ml ascorbic acid (Wako Pure Chemical, Osaka, Japan), 10 mM β‐glycerophosphate acid (Merck, Darmstadt, Germany).

### Cytotoxicity test

Primary BMCs were cultured into 96‐well plates at an initial number of 2.5 × 10^4^ cells in 100 μl per well for 48 hrs with LC or LP supplementation in α‐MEM. The control was prepared as well without liposomes. Then, the cells in each well were examined for a cytotoxic effect of those liposomes on cell survive using a cell proliferation and cytotoxicity assay kit (Cell Counting Kit ‐8; Dojindo Molecular Technologies, Inc., Kumamoto, Japan). WST‐8 formazan reduced by NADH produced in live cells were observed and quantified at a wave length of 450 nm using the Wallac microplate reader.

### Alkaline phosphatase/TRAP staining and activity assay

Culture cells exposed to liposomes were fixed with 4% formaldehyde (Wako Pure Chemical) after 1 week of culture in osteogenic medium. The cells were stained for alkaline phosphatase (ALP) followed by TRAP staining using the ALP/TRAP staining kit according to the manufacturer's protocols (Wako Pure Chemical) and analysed.

Similarly, the cells treated with liposomes followed by osteogenic medium for 1 week were lysed with 0.1% Triton X‐100 (Sigma‐Aldrich) and used for alkaline phosphatase activity (ALP) assay as mentioned before. The absorbance was recorded at 405 nm using a Wallac microplate reader (ARVO SX 1420 multilevel counter; Perkin Elmer Life Sciences, Waltham, USA). The collected data were normalized by the amount of DNA in each tested sample. The DNA standard used was salmon sperm DNA (F012; Funakoshi Co., Tokyo, Japan), and samples were stained with Hoechst33258 (343‐07961; Wako Pure Chemical) and observed at 325 nm using the Wallac microplate reader.

### RNA extraction and real‐time PCR

The mRNA levels of *Runx2*,* Dlx5*, and *Bmpr2* were quantitatively analysed. TRIzol reagent was used to extract RNA (Thermo Fisher Scientific, Waltham, USA) from cultured cells according to the manufacturer's protocol. cDNA was synthesized from 1 μg RNA using Super Script^®^III First‐Strand Synthesis Supermix for quantitative (or real time) reverse transcription polymerase chain reaction (qRT‐PCR) (Thermo Fisher Scientific, Waltham, USA). Real‐time RT‐PCR was performed with Power SYBR^®^ Green PCR Master Mix (Thermo Fisher Scientific) per manufacturer's protocol and processed using the 7300 real‐time PCR system (Applied Biosynthesis). The mouse primers designed for PCR reactions were as follows: *Dlx5*, forward: 5′‐AGGTGAGGATGGTGAATGGT‐3′ and reverse: 5′‐CTCCCCGTTTTTCATGATCT‐3′; *Runx2*, forward: 5′‐TGCTATTGCCCAAGATTTGC‐3′ and reverse: 5′‐GAGGGGGAAATGCCAAATAA‐3′; *Bmpr2*, forward: 5′‐GAAATCTCCAAGTGCCCAAA‐3′ and reverse: 5′‐GGTGTTGAGAAGCCTGAAGC‐3′; *Rank,* forward: 5′‐CGAGGAAGATTCCCACAGAG‐3′ and reverse: 5′‐CAGTGAAGTCACAGCCCTCA‐3′; *Oct3/4*, forward: 5′‐GTTGGAGAAGGTGGAACCAA‐3′ and reverse: 5′‐CCAAGGTGATCCTCTTCTGC‐3′;*Gapdh*, forward: 5′‐ACCCAGAAGACTGTGGATGG‐3′ and reverse: 5′‐CACATTGGGGGTAGGAACAC‐3′. The expression of the test genes was normalized to that of *Gapdh*, an internal standard.

### Collection of conditioned medium

Conditioned medium was prepared in BMSC culture by adding LC or LP (0.1 mg/ml) to α‐MEM or osteoblastic medium. Conditioned medium was collected after 72 hrs and filtered using a 0.22‐μm filter (Millex^®^‐GS filter unit 33 mm; Merck, Darmstadt, Germany). The medium was centrifuged at 1000 × *g* for 10 min. to remove cells and cell debris before use for culturing another batch of bone marrow cells for 1 week. The conditioned medium was changed after every three days. As a negative control, α‐MEM and osteogenic medium were prepared in the same way as the conditioned media.

### Isolation and quantification of exosomes

Exosomes were isolated from conditioned medium according to the protocol of ExoQuick‐TC (System Biosciences, Mountain View, CA, USA). α‐MEM and four different conditioned media such as α‐MEM with LC (LC‐conditioned medium), α‐MEM with LP (LP‐conditioned medium), osteogenic medium with LC (LC‐conditioned osteogenic medium) and osteoblastic medium with LP (LP‐conditioned osteogenic medium) were examined. For each 5 ml of collected medium, 1 ml of ExoQuick‐CT was added and incubated at 4°C overnight to precipitate exosomes, followed by centrifugation. The amount of exosomes from each sample was quantified by the EXOCET exosome quantification assay kit (System Biosciences). The colorimetric assay at 405 nm was used along with the standard curve for quantification.

### Western blot

Once exosomes were isolated, they were resuspended in 1× radioimmunoassay (RIPA) buffer [25 mM Tris‐HCl pH 7.6, 150 mM NaCl, 1% NP‐40, 1% sodium deoxycholate and 0.1% sodium dodecyl sulphate (SDS)] and 2× Laemmli buffer (4% SDS, 20% glycerol, 10% 2‐mercaptoethanol, 0.004% bromophenol blue and 0.125 M Tris‐HCl pH 6.8) as indicated in the ExoQuick‐TC protocol (System Biosciences). A standard SDS‐polyacrylamide gel electrophoresis (NuPAGE 4‐12% Bis‐Tris Gel 1.0 mm × 12 well; Invitrogen, USA) was performed using 120 μg protein lysates. The gel was transferred onto polyvinylidene fluoride (PVDF) membrane using the iBlot^®^ transfer stack (Thermo Fisher Scientific) and blocked for 30 min. (WesternBreeze^®^ Immunodetection System for mouse primary antibodies; Thermo Fisher Scientific, Waltham, USA) before incubation for anti‐RANK for 1 hr on a shaker (ab13918: 1 μg/ml). The secondary antibodies (WesternBreeze^®^ Immunodetection System for mouse primary antibodies, Novex^®^ by Life Technologies) were applied before colour development by chromogen for 1 hr.

### Liposomal injection into mouse femur

Upon *in vivo* surgery, each mouse was anaesthetized by intraperitoneal injection of pentobarbital at 60‐65 mg/kg (Somnopentyl^®^; Kyoritu Pharmatheutical Co., Ltd., Nara, Japan). For femur surgery, the skin over the knee region was cut open with a pair of surgical scissors 1 cm perpendicular to the body axis and exposed enough to observe the entire skeletal structure of the knee. A #40 K‐file was first inserted aside from meniscus into the femur, followed by gentle filing of a 1 cm‐deep cavity with # 60 K‐file such it did not penetrate the cortical bone and the other end of the femur. The cavity created in the femur was then filled with LC or LP (0.25 mg) using a 1‐ml syringe and a 26G needle. The same volume of serine solution was added to fill the cavity of the femur as a control. The knee was sutured, and mice were kept in a cage with water and food provided for the following 24 hrs.

### Immunohistochemistry

The mouse femur with a cavity created by K‐files was fixed in 4% formaldehyde for 48 hrs at 4°C and washed with running tap water for 5 hrs. Decalcification was performed with 10% EDTA by exchanging the solution after every 2–4 days until the tissue softened. The samples were dehydrated with ascending concentrations of ethanol, followed by clearing with xylene before embedding in paraffin (histoparaffin m.p. 56–58°C; Wako Pure Chemical). Five micrometre‐thick paraffin sections were made, de‐paraffinized and processed for immunohistochemistry. The section was blocked in G‐Block (Genostaff GB‐01; Genostaff Co., Ltd, Tokyo, Japan) at room temperature and incubated at 4°C overnight with primary antibodies, F4/80 (0.4 μg/ml) (ab6640 abcam^®^) and CD11b (1 μg/ml) (ab75476 abcam^®^), for macrophages and monocytes, respectively. The secondary antibodies were anti‐rabbit biotin (Dako E0432) and anti‐rat biotin (Dako E0468) for monocytes and macrophages, respectively. The sections were further reacted with streptavidin (Nichirei Biosciences 426062, Tokyo, Japan) for 5 min. at room temperature followed by DAB/H_2_O_2_ and haematoxylin and eosin (H & E) staining. The sections were sealed with Malinol 750cps^®^ (Muto Pure Chemicals, Tokyo, Japan) for observation.

### ALP and Alizarin red staining and ALP activity assay

After administration with liposomes, the mouse femoral bone marrow cells were flushed out for culture, seeded at a density of 3 × 10^5^ cell/well in 12‐well plates. The cultured cells were fixed with 4% formaldehyde (Wako Pure Chemical) and stained for alkaline phosphatase (ALP) using the ALP/TRAP staining kit as described before (Wako Pure Chemical) after 1–2 weeks. In addition to ALP staining, ALP activity assay was performed as described before. Calcium deposition was also examined after 3 weeks of incubation of the BMSC culture using 2 g Alizarin Red S (Sigma‐Aldrich), pH 4.1–4.3.

### RNA extraction and real‐time PCR

The mRNA levels of *Runx2*,* Dlx5* and *Bmpr2* were also quantitatively analysed. RNA was extracted directly from the cells flushed out after the administration of liposomes. The procedure and the primer sets were the same as described in previous section.

### Statistical analysis

All data were analysed as mean ± standard deviation (S.D.). Differences among conditions were calculated by one‐way analysis of variance (anova) using the SPSS software version 20 (IBM Corp. Armonk, NY, USA). A *p* value <0.05 was considered statistically significant.

## Results

### Cell survival after exposure to LC

The cytotoxicity test revealed that the liposomal carrier influenced cell viability as LP decreased the number of the bone marrow‐derived primary cells (Fig. 2H). On the other hand, efficacy of clodronate might mask such a liposomal inhibitory effect on cell proliferation as demonstrated in LC group (Fig. 2H).

### ALP/TRAP‐positive cells by LC

**Figure 1:**
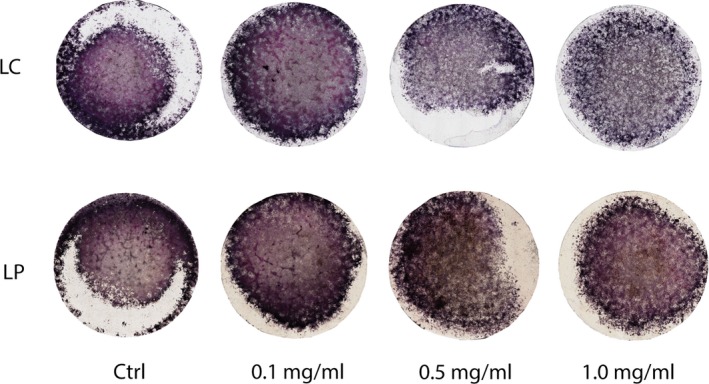
ALP/TRAP staining images of bone marrow mesenchymal stem cell (BMSC). Liposomal PBS (LP) and clodronate (LC) were added to BMSC culture for 24 hr at concentrations of 0.1, 0.5, and 1.0 mg/ml before switching to osteogenic medium. ALP (purple) and TRAP (pink). Control (Ctrl) received no liposomes.

Evaluation of the effects of LC on mouse BMSC cultures showed differences in colorimetric intensity for ALP and TRAP staining. Staining intensities of both ALP and TRAP decreased at higher concentrations of LC (0.5 and 1.0 mg/ml), but not at 0.1 mg/ml (Fig. 1). LC (0.1 mg/ml) did not alter the degree of staining; it even slightly enhanced the staining intensity compared to that of the control (Fig. 1). There was no change in staining intensity with liposomal PBS (Fig. 1).

**Figure 2:**
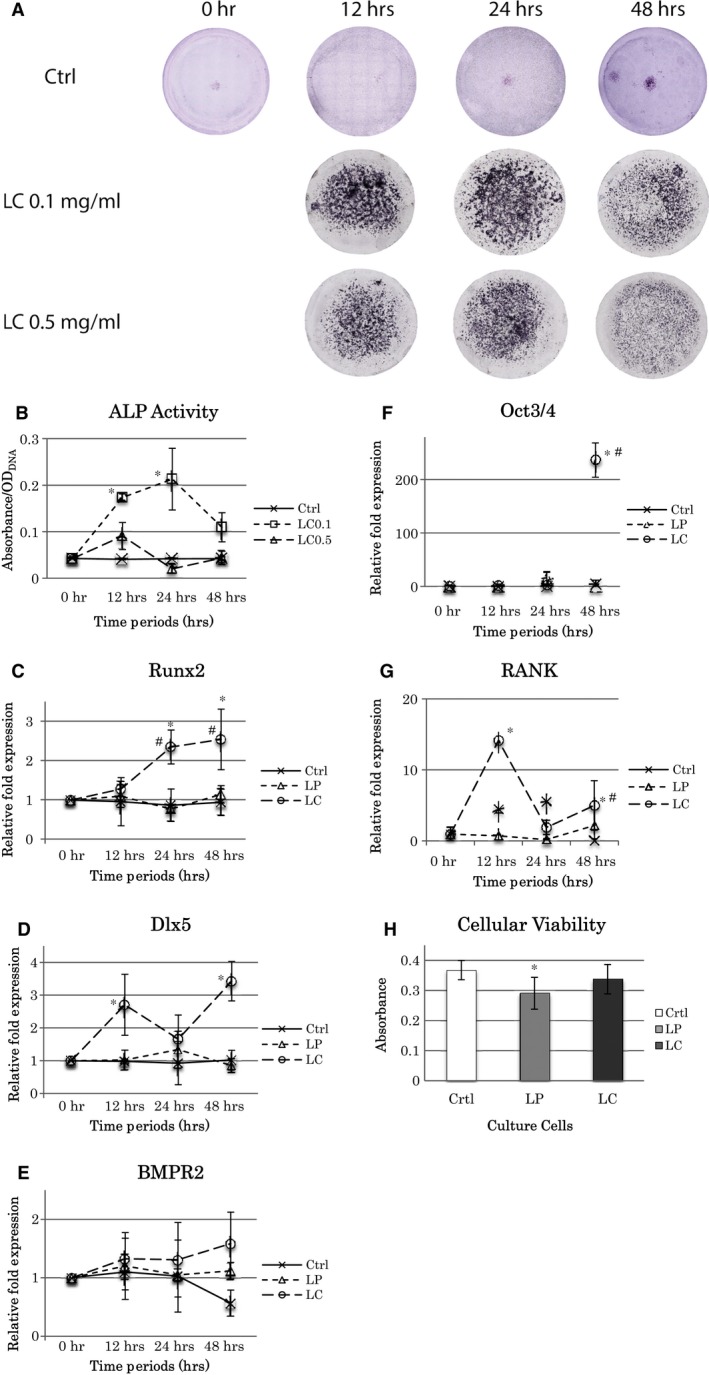
Effect of liposomal clodronate (LC) concentration and treatment time on BMSC culture. Cells were cultured for 12, 24 and 48 hr with 0.1 mg/ml LC or 0.5 mg/ml LC. Liposomal PBS (LP) treatment was performed as well. Control was cultured without liposomes for 0 to 48 hr. (**A**) ALP staining images of BMSC with 0, 12, 24 and 48 hr of LC (0.1 or 0.5 mg/ml) treatment in cell culture. (**B**) ALP activity analysis. *: significantly different with both Ctrl and LC0.5 at each time point (*P* < 0.05). (**C**–**G**) RT‐PCR analysis. The concentration of LC and LP was 0.1 mg/ml. (**C**) Runx2, *: significantly different with both Ctrl and LP at each time point (*P* < 0.05); #: significantly different from 12 hr (*P* < 0.05). (**D**) Dlx5, *: significantly different with Ctrl and LP at each time point (*P* < 0.05). (**E**) Bmpr2, no significant difference was observed. (**F**) Oct3/4, *: significantly different with both Ctrl and LP (*P* < 0.01); #: significantly increased from earlier time points (*P* < 0.05). (**G**) RANK, *: significantly different with both Ctrl and LP at each time point (*P* < 0.01). #: significantly increased from 24 hr (*P* < 0.05). (**H**) Cellular viability after LP and LC at 0.1 mg/ml treatment. *: significantly different with Ctrl (*P* < 0.05). All the statistical data are represented as the mean ± SD by one‐way anova (*n* = 3).

Furthermore, ALP staining varied with the duration and concentration of LC in the BMSC culture. The staining was more at both 12 and 24 hrs with 0.1 mg/ml of LC compared to those in the other conditions (Fig. 2A), which was coincident with a significant increase in ALP activity detected at the same time‐points with 0.1 mg/ml of LC (Fig. 2B). Although there was a decrease in ALP activity at 48 hrs with 0.1 mg/ml LC, lower concentration of LC caused enhancement in ALP activity than higher concentration of LC throughout the time periods tested (Fig. 2B). There was no significant difference between 12‐ and 24‐hr treatment with 0.1 mg/ml of LC.

### Osteoblast‐specific gene expression with LC

Osteogenic gene expression changed after LC treatment of the BMSC culture. Real‐time RT‐PCR demonstrated significant increase in Runx2 with application of 0.1 mg/ml LC for 24 and 48 hrs, whereas LP did not change Runx2 level over time (Fig. 2C). On the contrary, the lowest LC administration for 12 hrs did not show significant difference compared to the control (Fig. 2C). The pattern of Dlx5 mRNA expression was similar to that of Runx2, with highest expression after 48 hrs with LC and no effect with LP (Fig. 2D). Even though mRNA levels at 24 hrs with LC were higher than those of the control and LP groups, it was not statistically significant (Fig. 2D). Bmpr2 levels did not differ between the LC and LP treatments at any time period (Fig. 2E). Furthermore, mRNA expression level of RANK, as a receptor associated with osteoclast differentiation by direct contact with an osteoblast cellular membrane ligand, RANKL, was elevated by LC at 12 and 48 hrs (Fig. 2G), and there was a spike of Oct3/4, a stem cell marker, gene expression by LC at 48 hrs (Fig. 2F).

### Osteogenesis and exosome release by a conditioned medium exposed to LC

**Figure 3:**
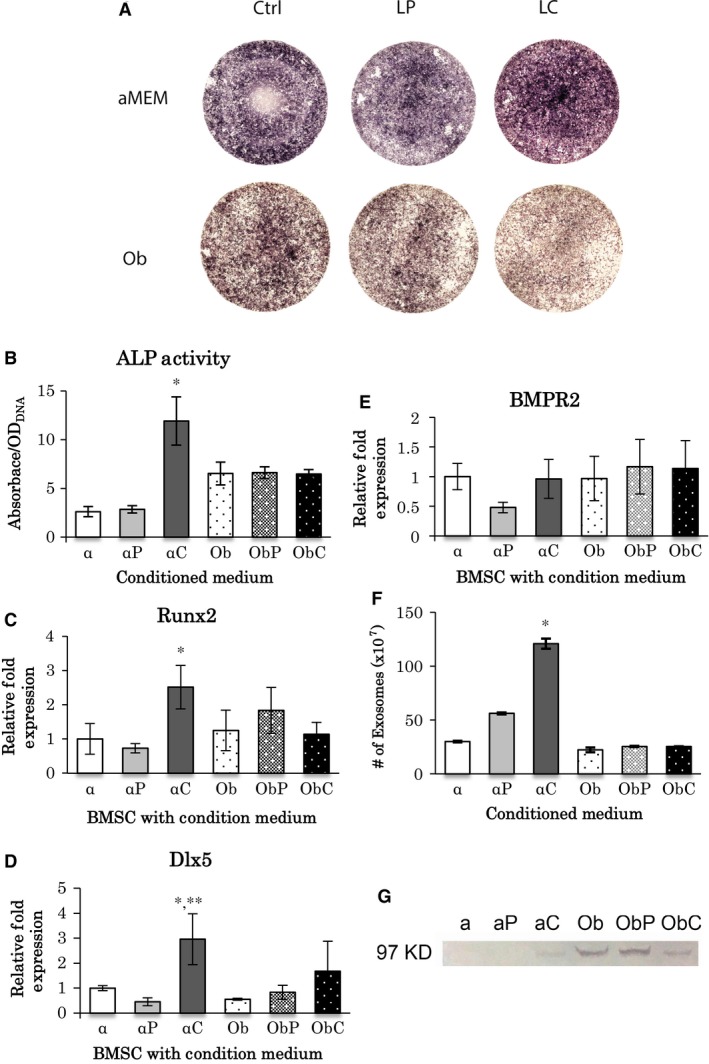
Analysis of conditioned‐medium after LC treatment of BMSC culture. (**A**) ALP staining of BMSCs cultured in conditioned media for 1 week. Conditioned‐medium was made in α‐MEM or osteogenic medium (Ob) containing either LP or LC (0.1 mg/ml). The control did not contain liposomes. (**B**) ALP activity analysis in α‐MEM (α), LP‐conditioned‐medium (αP), LC‐conditioned medium (αC), osteogenic medium (Ob), LP‐conditioned osteogenic‐medium (ObP), and LC‐conditioned osteogenic‐medium (ObC). *: significantly different among all treatments (*P* < 0.05, *n* = 3). (**C**–**E**) Real time RT‐PCR of BMSCs cultured in conditioned‐media. mRNA expressions of Runx2 (**C**), Dlx5 (**D**) and BMPR2 (**E**) were examined (*n* = 3). (**C**) *: αC versus α, αP and ObC (*P* < 0.05); (**D**) *: αC versus α and ObC (*P* < 0.05); **: αC versus αP, Ob and ObP (*P* < 0.01). (**F**) The number of exosomes found in conditioned‐media. *: significantly different with all treatments (*P* < 0.05, *n* = 5). (**G**) Detection of RANK using western blot analysis in conditioned‐media. The size for RANK is 97 KDa. All statistical data are represented as mean ± SD by one‐way anova.

Intense ALP staining was observed with LC‐conditioned medium (Fig. 3A). The change in ALP staining is consistent with ALP activity, and the LC‐conditioned medium showed significantly high activity among all media (Fig. 3B). The comparison of LP or LC‐conditioned osteogenic medium to LP or LC‐conditioned medium showed that conditioned osteogenic media had slightly reduced overall ALP staining intensity (Fig. 3A). Furthermore, BMSCs cultured in the LC‐conditioned medium showed the highest mRNA levels of Runx2 and Dlx5 than those in the other media (Fig. 3C,D); however, Bmpr2 mRNA level was not significantly altered by the medium (Fig. 3E).

A significantly large number of exosomes (120 × 10^7^) was observed in the LC‐conditioned medium (Fig. 3F). In contrast, other conditioned media did not contain significant amount of exosomes (ranging from 30 to 60 × 10^7^), and there were no significant differences among them (Fig. 3F). Subsequently, RANK was detected by Western blot analysis. An expected band at 97 kD was observed in both types of osteogenic‐conditioned media as well as osteogenic medium (control) and LC‐conditioned medium (Fig. 3G). The band signals weakened with LC‐conditioned medium and LC‐conditioned osteogenic medium. Interestingly, RANK was detected only in the LC‐conditioned medium but not in conditioned medium prepared in α‐MEM (Fig. 3G).

### Reduction in macrophage and monocyte numbers in mouse femur promoted osteogenesis by LC

**Figure 4:**
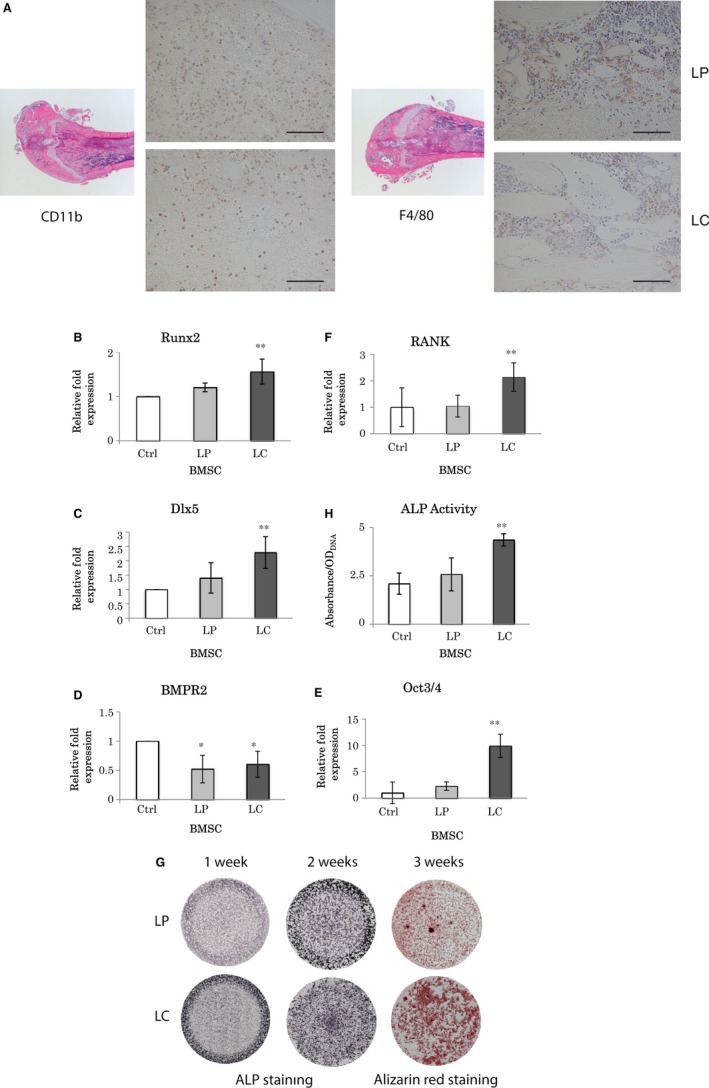
Liposomal clodronate injection into mouse femur. (**A**) H&E staining and immunohistochemistry on mouse femur injected with LP or LC (50 μl). Immunohistochemistry detecting CD11b (monocytes) and F4/80 (macrophages) on paraffin‐embedded sections. Scale bar = 100 μm. (**B**–**F**) Real time RT‐PCR analysis on mouse BMSCs flushed out from femur 24 hr after LP and LC injection. The same volume of saline solution was injected into the femur as control. (**B**) Runx2 and (**C**) Dlx5, **: significantly different with LP (*P* < 0.05) and Ctrl (*P* < 0.01). (**D**) BMPR2, *: significantly different with Ctrl (*P* < 0.05). (**E**) Oct3/4, **: significantly different with Ctrl and LP (*P* < 0.01). (**F**) RANK, **: significantly different with Ctrl and LP (*P* < 0.05). (**G**) ALP and Alizarin Red staining images. ALP staining at 1 and 2 weeks, and Alizarin Red staining at 3 weeks. Comparison between LP (upper low) and LC (lower low). (**H**) ALP activity analysis on mouse femur BMSC 24 hr after liposomal injections. **: significantly different with LP and Ctrl (*P* < 0.01). All statistical data are represented as mean ± SD by one‐way anova (*n* = 4).

H & E staining of the femur at lower magnification confirmed the presence of an access cavity created for LP or LC injection in the centre of the femoral head and no other penetrations in the femur (Fig. 4A). Signals for both F4/80 and CD11b were localized and exaggerated in LP‐administered femur than in the LC injection group (Fig. 4A). LC decreased the numbers of macrophages and monocytes in femur.

Real‐time RT‐PCR results revealed that mRNA levels were significantly elevated 1.6‐fold for Runx2 and 2.3‐fold for Dlx5 by LC administration (Fig. 4B,C), whereas that of Bmpr2 was halved by both LP and LC (Fig. 4D). Furthermore, Oct3/4 and RANK gene expression levels were increased 9.9‐ and 2.1‐folds by LC, respectively (Fig. 4E, F).

LC femoral administration enhanced both ALP activity and mineralization (Fig. 4G,H). In contrast, mineralization was not strong in LP‐injected femurs (Fig. 4G). Highest ALP activity with LC was consistent with the highest staining for ALP in the same treatment group (Fig. 4H). The presence of LC elevated ALP activity, calcification level and expressions of osteogenic genes, whereas it reduced the numbers of macrophages and monocytes in mouse BMSCs (Fig. 4A–H).

## Discussion

Systemic or local administration of bisphosphonates have been widely used in clinical treatments not only to decelerate osteoporotic bone resorption, but also to regenerate and increase bone mass in a damaged condition such as bone distraction 25, 26, 27. Few reports of accelerated ossification in healthy animals by bisphosphonates exist 28, 29. Hayden *et al*. 15 recently demonstrated an effect of bisphosphonates on osteoblasts and osteoclasts with or without their coculture. Although the mechanism by which osteoclast maturation was stimulated is a well‐known signalling pathway involving the binding of osteoblast‐expressed RANK ligand to a membrane receptor, RANK, on osteoclast precursors 30, it is not known whether osteoblasts can be differentiated and activated, directly or indirectly, by osteoclasts, and if the two types of cells have a synergistic effect on their maturation and function. The present study investigated microvesicle‐mediated osteoblast differentiation using liposomal clodronate administration both *in vitro* and *in vivo*.

In BMSC culture, LC at the higher doses of 0.5 and 1.0 mg/ml reduced both TRAP‐positive and ALP‐positive cells, which represented the inhibitory effect of LC on osteoclastic function and its desensitizing 31, 32, 33 or inactivating effect on osteoblastic function; however, LC concentrations as low as 0.1 mg/ml enhanced both TRAP‐positive and ALP‐positive staining. Furthermore, transcription of osteoblast‐related genes, *Dlx5* and *Runx2*, increased with the lowest concentration of LC (0.1 mg/ml) after 48 hrs culture. Expression of RANK, osteoclastgenesis‐related gene was elevated by LC supplemented in the culture media at 12 and 48 hrs. Although the mechanism by which bisphosphonates are endocytosed into osteoclast precursors is well known to inhibit not differentiation but function of osteoclasts, the stimulated gene expression of RANK cellular membrane receptor seemed compensated for released exosome‐RANK, which could be observed also in the femoral injection. Interestingly, mRNA level of Oct3/4, a major stem cell marker, increased significantly by LC treatment at 48 hrs, suggesting stimulatory effects of LC on both expansion of stem cells and differentiation of osteoblasts. It is likely that clodronate performs dual reciprocal roles, which are inhibitory and stimulatory to both osteoclast generally and osteoblast differentiations as a new knowledge. On the contrary, the femoral injection of 0.25 mg LC resulted in decrease in the number of macrophages and monocytes in the femoral medulla, confirming its inhibitory effects on proliferation of monocytes and macrophages, which corroborates the results of other studies regarding the regulatory effect of bisphosphonate administration 19, 34, 35; however, strong ALP activity and staining were observed, accompanied by prominent mineralization, in the LC‐injected femurs. Vi *et al*. 5 suggested that macrophages are crucial for maintaining bone homeostasis and promoting fracture repair by enhancing the differentiation of mesenchymal progenitors, whereas bone union was impaired and calluses were smaller with less bone and more fibrotic tissue deposition when macrophages were depleted during fracture repair. Under these depletion conditions, the differentiated forms of macrophages, including osteoclast precursors and osteoclasts, could have been the targets for functional abandonment in addition to the macrophages. However, our femoral injection of LC demonstrated enhancement of Oct3/4 gene expression, which could imply increase in stem cells for cell differentiation as well as in the cell culture study. In fact in the current study, LC administration in cell culture and bone medulla resulted in acceleration of osteogenesis by elevating the ALP‐positive cell number and mineral deposition. However, conditional knockout models or cell culture studies using osteoblastic cell lines would have been appropriate to investigate the signalling pathways of clodronate to osteoblasts and preosteoblasts. Considering the mutual elevation of osteoblast and osteoclast differentiation in our *in vitro* study, we suggested that the acceleration of osteoblast differentiation in the LC‐injected femoral cavities had not been promoted by macrophages and monocytes but was proportionate to the number or intensity of multinuclear TRAP‐positive cells, which might include differentiated precursors of osteoclasts.

Generally, the presence of BMP‐2 in osteogenic medium enhances BMSC commitment to osteoblast differentiation and the expression of its receptors BMPR1A and BMPR2. However, the lower levels of Bmpr2 mRNA in LC‐administered femurs, along with the increase in osteogenic activity, might not be related to signalling through BMPR2, and BMPR2‐mediated signalling might have been compensated in the body. Exosomes are known to enhance ALP activity, cell proliferation and expression of bone‐related mRNA *in vitro* 21, 36, 37. Furthermore, they stimulate bone regeneration and angiogenesis *in vitro* 38 and *in vivo* 17, 18, 23. In the present study, LC in the culture media induced the generation of large numbers of exosomes, especially in a conditioned medium of the non‐osteogenic environment. And it was more obvious and favoured in the non‐osteogenic environment. Thus, LC‐stimulated microvesicle‐like exosomes might play a role in transmitting signals for osteogenesis in a BMP‐poor condition. However, the numbers of macrophages and monocytes decreased upon LC administration *in vivo*, suggesting that other cell types might contribute to exosome production. Although we did not evaluate the response of osteoclasts and their precursors to the femoral administration of LC, our cell culture study demonstrated that TRAP‐positive cells were enhanced by LC supplementation. Therefore, osteoclastic cells would be a candidate cargo for exosomes, which should be examined in future.

The type of molecules that can be secreted and transferred by exosomes and the nature of the major vesicles involved at injury sites upon bisphosphonate administration are not known. RANKL‐to‐RANK intermembrane signal transmission is well known to trigger and stimulate osteoclast differentiation 24, 39, 40, 41; however, whether osteoclasts may contribute to osteoblast differentiation is unknown. Here, we examined whether RANK could be secreted and transferred by exosomes for inducing RANK‐related osteoblast differentiation. Soluble RANK was observed only in LC‐conditioned medium, and not in control and LP‐conditioned media, by Western blot analysis. Although the level of RANK in LC‐conditioned medium was lower than that obtained in other osteogenic media, it is clear that LC stimulated secretion of soluble RANK in exosomes. Further, higher levels of soluble RANK in the osteogenic media were possibly due to the presence of dexamethasone, as exosomes were not observed in those culture media, suggesting that exosomes may not be the only molecules for transferring soluble RANK. It is noteworthy that RANK mRNA expression was stimulated by LC treatment. Taking together, it suggests clodronate might induce not only released exosome‐RANK but also gene expression of RANK in the cultured bone marrow cells, possibly osteoclast precursors, macrophages and monocytes although the number of macrophages and monocytes was decreased. However, it remains unclear whether RANK is intimately involved in osteoblast differentiation as observed in this study.

The present study suggested a dual role of liposomal clodronate in regulation of osteoblast and osteoclast differentiation and a new possible mechanism for osteoblast differentiation through exosome‐enclosed soluble RANK. In conclusion, liposomal clodronate enhanced osteoblast differentiation of mouse MSCs both *in vitro* and *in vivo*.

## Conflict of interests

The authors confirm that there are no conflict of interests.
